# A systematic investigation of the maximum tolerated dose of cytotoxic chemotherapy with and without supportive care in mice

**DOI:** 10.1186/s12885-017-3677-7

**Published:** 2017-10-16

**Authors:** Wayne J. Aston, Danika E. Hope, Anna K. Nowak, Bruce W. Robinson, Richard A. Lake, W. Joost Lesterhuis

**Affiliations:** 1 National Centre for Asbestos Related Diseases, University of Western Australia, 5th Floor, QQ Block, 6 Verdun Street, Nedlands, WA 6009 Australia; 2 Faculty of Health and Medical Science, The University of Western Australia, 35 Stirling Highway, Crawley, WA 6009 Australia; 3 Department of Medical Oncology, Sir Charles Gairdner Hospital, Nedlands, WA 6009 Australia

**Keywords:** Chemotherapy, Mice, Dose optimization, Maximum tolerated dose, MTD, Supportive care, Cancer

## Abstract

**Background:**

Cytotoxic chemotherapeutics form the cornerstone of systemic treatment of many cancers. Patients are dosed at maximum tolerated dose (MTD), which is carefully determined in phase I studies. In contrast, in murine studies, dosages are often based on customary practice or small pilot studies, which often are not well documented. Consequently, research groups need to replicate experiments, resulting in an excess use of animals and highly variable dosages across the literature. In addition, while patients often receive supportive treatments in order to allow dose escalation, mice do not. These issues could affect experimental results and hence clinical translation.

**Methods:**

To address this, we determined the single-dose MTD in BALB/c and C57BL/6 mice for a range of chemotherapeutics covering the canonical classes, with clinical score and weight as endpoints.

**Results:**

We found that there was some variation in MTDs between strains and the tolerability of repeated cycles of chemotherapy at MTD was drug-dependent. We also demonstrate that dexamethasone reduces chemotherapy-induced weight loss in mice.

**Conclusion:**

These data form a resource for future studies using chemotherapy in mice, increasing comparability between studies, reducing the number of mice needed for dose optimisation experiments and potentially improving translation to the clinic.

**Electronic supplementary material:**

The online version of this article (10.1186/s12885-017-3677-7) contains supplementary material, which is available to authorized users.

## Background

Cytotoxic chemotherapy still forms the basis of systemic therapy for many cancers. Treatment plans typically consist of repeated cycles of chemotherapy at as high a dose as possible, without causing unacceptable toxicity, the maximum tolerated dose (MTD). Maximum drug doses are determined in dose escalating phase I clinical trials until reaching an appropriate balance between efficacy and toxicity [1]. In contrast, preclinical studies usually employ doses based on convention within a research group, on published studies that may or may not have reported optimisation experiments or on quick optimisation steps in which the methods are varied [2, 3]. In addition, while in clinical studies further dose escalation is allowed by extensive supportive care measures such as intravenous hydration, anti-emetics, antihistamines and corticosteroids; this is usually lacking in animal studies [2]. Together, this may result in both the use of sub-therapeutic dosages and excess use of animals.

There is a clear dose-response relationship between chemotherapy and tumour regression in preclinical studies [4] and in the clinical setting [5]. Furthermore, there are many secondary antitumour effects that depend on chemotherapy dose, for example immune stimulatory potential of dendritic cells [6]; production of IL-17 by peripheral blood and splenic CD4^+^ T cells [7]; antiangiogenic effects [8, 9] or depletion of regulatory T cells [10–13]. Therefore, the dose used in preclinical studies may significantly affect translation into clinical trials [14]. Human MTDs are often well predicted by animal studies. A meta-analysis of the preclinical and subsequent clinical development phases of 25 cancer drugs showed that rodent toxicology generally provided a safe and reliable way of assessing starting dosages in humans and adequately predicted potential side effects [15]. It is reasonable then that preclinical studies should make use of MTD regimes. However, to our knowledge, there has not been a systematic study done to determine the MTD for chemotherapeutics from each of the canonical classes. As part of the clinical development pathway LD50 values (median lethal dose) have often been determined, giving some indication of where the MTD will be. However, many of these studies were done decades ago in mouse strains that are often no longer used in cancer research, while the tolerability to chemotherapy varies considerably between mouse strains [16–18]. This compromises the extrapolation of those MTDs to currently standard mouse strains. We therefore aimed to create a murine cancer chemotherapy MTD resource in BALB/c and C57BL/6 mice, which would both reduce individual dose optimising investigations and allow standardized dosing strategies in preclinical cancer research. We chose weight loss and clinical score (Additional file 1: Table S2) as endpoints, because in previous murine studies weight loss was by far the most common dose-limiting toxicity (81%), followed by clinical signs, such as neurotoxicity or diarrhoea [15]. This allowed us a straightforward way of assessing toxicity that we think is universally relevant. We also tested whether the single-dose MTD could be readily extrapolated to repeated cycles and whether there were differences in MTDs between mouse strains. Lastly, because the corticosteroid dexamethasone and the 5-hydroxytryptamine 3 (5-HT_3_) receptor antagonist ondansetron are commonly administered in conjunction with chemotherapy to reduce nausea and anorexia in patients, we determined the effect of these drugs on the MTD in mice.

## Methods

### Mice

Female BALB/c and C57Bl/6 J mice were obtained from the Animal Resources Centre (Murdoch, Western Australia) and housed under specific pathogen free (SPF) conditions (M-block Animal Facility and Harry Perkins Bioresources Facility, Queen Elizabeth II Medical Centre, The University of Western Australia). Mice were between 8 and 10 weeks of age for these studies. All experiments were conducted according to the University of Western Australia Animal Ethics Committee approvals (Protocols RA/3/100/1139, RA/3/100/1217) and the Harry Perkins Institute for Medical Research Animal Ethics Committee (AEC029–2015) and the code of conduct of the National Health and Medical Research Council of Australia. Mice were fed rat and mouse cubes (Speciality Feeds, Perth, WA Australia) and housed on aspenchipsAB3 bedding (Datesand, Manchester UK). The animal facility temperature was kept between 21 °C and 22 °C.

### Chemotherapy dosing

The following chemotherapies were used in these studies: 5-fluorouracil (5-FU), bleomycin, cisplatin, cyclophosphamide (CY), docetaxel, doxorubicin, etoposide phosphate, gemcitabine, irinotecan, vinorelbine and were obtained from the pharmacy department at Sir Charles Gardiner Hospital, Perth, Australia. Further details are available in Additional file 1: Table S1. All mice were dosed intraperitoneally (i.p) using a 29G insulin syringe. Chemotherapy was prepared and diluted under sterile conditions in either phosphate buffered saline (PBS) or 0.9% sodium chloride as per manufacturer’s instructions. Where possible, chemotherapy was made to a dilution whereby a 20 g mouse would receive a 100 μl i.p injection.

### Determination of MTD

To determine the MTD, we used two endpoints: weight loss and clinical score. Clinical signs [15, 19] were scored by observing activity, appearance and body condition with a maximum of 2 points going to each (0, normal; 1 slight deviation from normal; 2, moderate deviation from normal, Additional file 1: Table S2). The starting doses were based on literature review, taking a dose that was reportedly safe to administer in one or more publications. Doses were escalated incrementally in steps of not more than 50% of the original dose until any mice met the primary endpoint of either >15% weight loss or reached a clinical score > 2. When either of these endpoints was met, dose escalation was ceased and the prior dose was set as the MTD. Euthanasia criteria included weight loss ≥20% or clinical score ≥ 3, and in such cases the previous identified safe dose was set as the MTD. Mice were monitored daily until both weight and clinical condition returned to baseline.

### Repeated cycles of chemotherapy

To determine the effect of repeated chemotherapy dosing, BALB/c mice were dosed i.p. with either 4 mg/kg or 6 mg/kg cisplatin or 10 mg/kg vinorelbine. Once mice had recovered to 100% of their starting weight or a clinical score of 0, a second MTD was given. Dosing was repeated for a total of 3 cycles of dosing.

### Effect of supportive care on chemotherapy-induced weight-loss

BALB/c mice were dosed i.p. with cisplatin at MTD in combination with dexamethasone (DBL dexamethasone sodium phosphate, Hospira, Mulgrave VIC Australia), ondansetron (Ondansetron-Claris, Claris, Burwood NSW Australia) or both. Dexamethasone and ondansetron were diluted with sterile water for injection and dosed on the day of chemotherapy administration and for 3 days post chemotherapy. Dose titrations were conducted for dexamethasone while ondansetron was dosed at the commonly reported dose of 1 mg/kg in mice [20].

### Statistical analysis

To determine a difference of 15% loss from starting weight, with a standard deviation of 5%, assuming an α of 0.05 and P 0.80, the sample size was calculated to be three mice per group using a paired t-test analysis.

Statistical significance of the weight loss nadir in mice treated with chemotherapy with or without supportive care was determined using a student’s t-test.

## Results

### Determination of MTD of selected chemotherapeutics

**Fig. 1:**
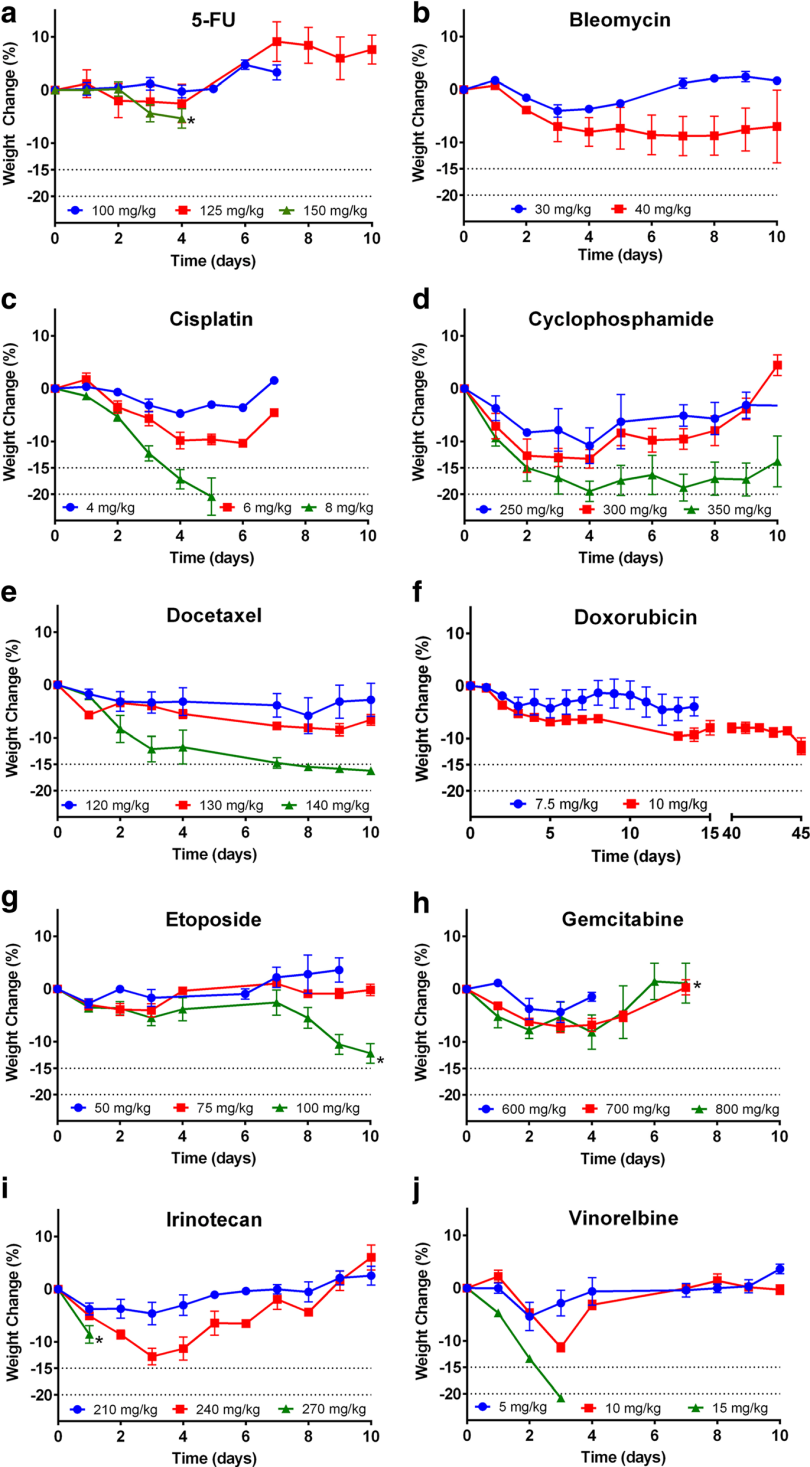
Maximum Tolerated Dosages for Chemotherapy in BALB/c mice. Body weight as a percentage of original of mice dosed with (**a**) 5-FU, (**b**) bleomycin, (**c**) cisplatin, (**d**) cyclophosphamide, (**e**) docetaxel, (**f**) doxorubicin, (**g**) etoposide, (**h**) gemcitabine, (**i**) irinotecan, (**j**) vinorelbine. *Clinical score ≥ 3. #Weight/clinical score did not return to baseline. Depicted are mean weights as percentage of starting weights with SEM (*n* = 3 mice/group)

We first determined the MTD of a range of chemotherapeutics from each class in BALB/c mice. We found a clear correlation between dose and weight loss and/or clinical score for all chemotherapeutics (Fig. 1). The established MTDs for a single dose were: 5-FU 125 mg/kg, bleomycin 30 mg/kg, cisplatin 6 mg/kg, cyclophosphamide 300 mg/kg, docetaxel 130 mg/kg, doxorubicin 7.5 mg/kg, etoposide 75 mg/kg, gemcitabine 700 mg/kg, irinotecan 240 mg/kg and vinorelbine 10 mg/kg. These dosages along with those commonly reported in the literature can be found in Table 1. Weight loss profiles differed between chemotherapeutics. The majority of drugs caused acute weight loss that returned to baseline within 10 days, with the exception of doxorubicin at 10 mg/kg, which led to an extended period (46 days) of weight loss. While this was not more than our endpoint of 15%, the fact that it did not return to baseline led us to set the previous dose of 7.5 mg/kg as the MTD. While weight loss was the primary dose-limiting toxicity for most chemotherapeutics, 5-FU, etoposide, gemcitabine and irinotecan endpoints were based upon clinical score. With gemcitabine, for example, mice became lethargic within minutes of administration. Although the clinical score improved after several hours, at single doses above 700 mg/kg, it remained at or above 3 for an extended period and so this dose was determined as the MTD.

**Table 1:** Chemotherapy Dosing. Comparison of the reported LD50 in the literature, MTDs determined in this study, common murine i.p. in vivo dosages and clinical dose

Chemotherapy	Class	Reported LD50 in Literature [49]	Single dose MTD	Common murine in vivo single dose	Clinical Dose*
5-Fluorouracil	Antimetabolite	100 mg/kg	125 mg/kg	50–60 mg/kg [35, 61, 62]	71 mg/kg divided over 2 days (a bolus of 400 mg/m^2^ plus continuous infusion of 2400 mg/m^2^) [63]
Bleomycin	Antitumour antibiotic	35 mg/kg	30 mg/kg	15 mg/kg [64–66]	30 mg (irrespective of weight) [67]
Cisplatin	Platinum compound	6.6 mg/kg	6 mg/kg	5–6 mg/kg [34, 38, 39]	2.5 mg/kg (100 mg/m^2^) [68]
Cyclophosphamide	Alkylating agent	420 mg/kg	300 mg/kg	200 mg/kg [30, 69, 70]	60 mg/kg/day for 2 days [71]
Docetaxel	Taxane	156 mg/kg IV	130 mg/kg	60–80 mg/kg [31–33]	2.5 mg/kg (100 mg/m^2^) [72]
Doxorubicin	Anthracycline	10.7 mg/kg	10 mg/kg	2–12 mg/kg [22–25]	1.9 mg/kg (75 mg/m^2^) [73]
Etoposide	Topoisomerase inhibitor	64 mg/kg	75 mg/kg	50 mg/kg [74–76]	5 mg/kg (200 mg/m^2^) [77]
Gemcitabine	Antimetabolite	2000 mg/kg [78]	700 mg/kg	120 mg/kg [35, 36, 79]	25 mg/kg (1000 mg/m^2^) [80]
Irinotecan	Topoisomerase inhibitor	177 mg/kg	240 mg/kg	59–100 mg/kg [81–83]	8.9 mg/kg (350 mg/m^2^) [84]
Vinorelbine	Vinca Alkaloid	26 mg/kg	10 mg/kg	10 mg/kg [40–42]	0.63 mg/kg (25 mg/m^2^) [85]

*Clinical dosages in patients are usually given as mg/m^2^, in those cases the amount of mg/kg was calculated based on a person of 1.9m^2^ (i.e. a person of 1.75 m height and a weight of 75 kg) as: [dose in mg/m2] × 1.9m^2^/75kg. Where clinical doses vary between indications, the highest dose is given with the appropriate reference. Note that clinical dosing is always in the context of repeated cycles

### Reproducibility of MTD between mouse strains

**Fig. 2:**
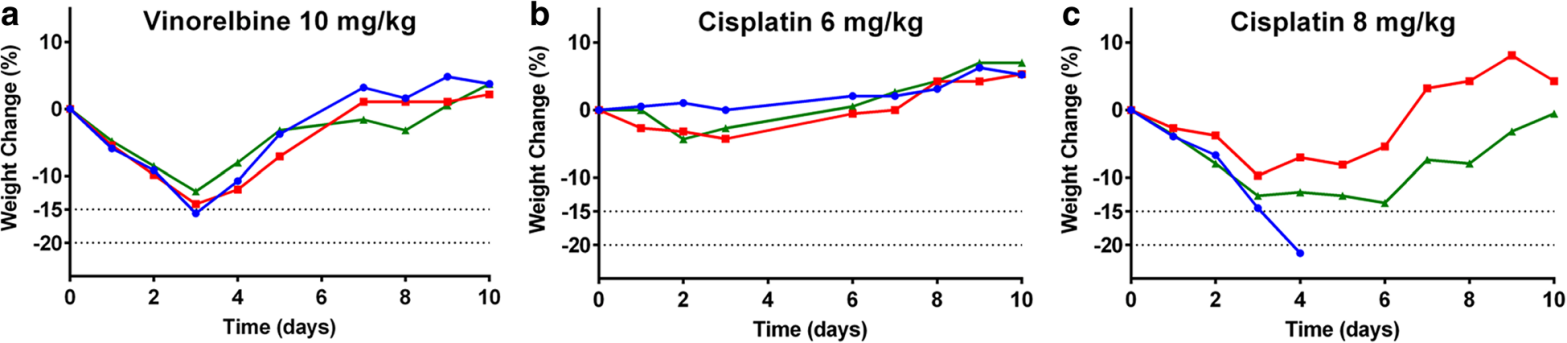
Maximum tolerated doses in C57BL/6 J mice. Individual body weights as a percentage of original for C57BL/6 J mice (*n* = 3 per group) treated with (**a**) vinorelbine 10 mg/kg, (**b**) cisplatin 6 mg/kg or cisplatin 8 mg/kg (**c**)

To assess the reproducibility of the MTD across mouse strains, we repeated dosing of two chemotherapeutics from distinct classes (vinca alkaloids and platinum-based compounds) in the C57BL/6 J strain (Fig. 2). We found similar weight loss in C57BL/6 J mice for vinorelbine at the BALB/c MTD of 10 mg/kg (Fig. 2a). Cisplatin at the BALB/c MTD of 6 mg/kg showed slightly less severe weight loss in the C57BL/6 J strain (Fig. 2b). However, when cisplatin dose was increased to 8 mg/kg (Fig. 2c), one of the three mice exceeded the 20% weight loss cut-off. The MTD for both tested chemotherapeutics was therefore similar for both strains.

### Repeated cycles of MTD chemotherapy

**Fig. 3:**
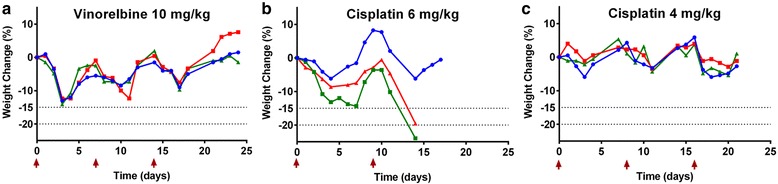
Effect of repeated cycles of chemotherapy given at MTD. Individual body weights as a percentage of starting weight for BALB/c mice (*n* = 3 per group) treated with repeated cycles of (**a**) vinorelbine 10 mg/kg, (**b**) cisplatin 6 mg/kg and (**c**) cisplatin 4 mg/kg. Doses were repeated when mice recovered to 100% of their starting weight. Arrows indicate dosing of respective chemotherapeutics. For vinorelbine this was day 0, 7, 14; for cisplatin 6 mg/kg this was day 0, 9; for cisplatin 4 mg/kg this was day 0, 8, 16

Since patients receive multiple courses of chemotherapy in the clinic and because toxicity to chemotherapeutics can be cumulative, we determined the effect of repeated dosing of chemotherapy at MTD. We gave 3 cycles at the single-dose MTD of 6 mg/kg for cisplatin and 10 mg/kg for vinorelbine. Vinorelbine was well tolerated at 10 mg/kg without any cumulative weight loss or deterioration in clinical score after repeated cycles (Fig. 3a). With repeat cisplatin administration at the single dose MTD, weight loss exceeded 20% in the second dosing cycle, (Fig. 3b). A lower dose of 4 mg/kg cisplatin was well tolerated and 3 cycles could be given without additional weight loss (Fig. 3c).

### Effect of supportive care on chemotherapy-induced weight loss

**Fig. 4:**
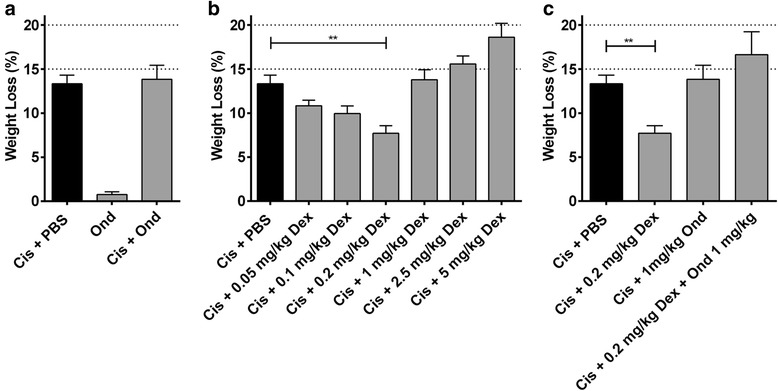
Effect of supportive treatment on cisplatin-induced weight loss. Individual maximum body weight loss as compared to starting weight for mice treated with cisplatin at MTD with or without ondansetron (**a**). Maximum weight loss shown for cisplatin 6 mg/kg plus dexamethasone at varied dosages (**b**) and cisplatin 6 mg/kg plus ondansetron 1 mg/kg and dexamethasone 0.2 mg/kg (**c**). Depicted are mean weight loss with SEM, *n* = 3 for all groups. **p < 0.01

In the clinical setting extensive supportive care measures are used so that higher doses can be tolerated. We therefore investigated the effect of supportive care with dexamethasone and ondansetron on both weight loss and clinical score of mice (Fig. 4). Ondansetron is a serotonin 5-HT_3_ receptor antagonist that is used as an anti-emetic to prevent chemotherapy-induced nausea and vomiting. Ondansetron was not effective in reducing weight loss induced by cisplatin (Fig. 4a). Dexamethasone, a corticosteroid also used in patients as an anti-emetic, was dose titrated and showed that higher dosages led to greater weight loss than cisplatin alone, however, doses below 1 mg/kg were effective at countering chemo induced weight loss with 0.2 mg/kg the most effective dose (Fig. 4b). In patients, ondansetron is often combined with dexamethasone to reduce nausea and vomiting, even in situations where ondansetron alone is not effective [21]. We tested the combination in mice and found that the benefit of dexamethasone was lost when ondansetron was added into the treatment regimen (Fig. 4c).

## Discussion

Chemotherapy administration to patients is governed by strict guidelines relating to dosage and scheduling as determined in dose-optimising phase I studies. Yet these same standards are often not applied to in vivo preclinical studies [2, 3]. When we searched the literature for chemotherapy dosages in order to inform related studies, we found that unlike the clinical situation, dosages varied widely between studies. For example, doxorubicin is reportedly used at dosages varying between 2 and 3 mg/kg [22, 23] and 10–12 mg/kg [24, 25]. In addition, in some studies, doxorubicin is given intratumourally [26–28], which may provide interesting mechanistic information, but does compromise translatability. A further discrepancy is with the definition of low and high dose chemotherapy. Low-dose cyclophosphamide has often been studied in regards to its capacity to deplete regulatory T cell (Treg) [29]. However, the concentrations used are quite varied, with doses ranging from 30 to 200 mg/kg, even though it has been shown that specific depletion of Tregs occurs at 20 mg/kg but not at 200 mg/kg [30]. Thus this unclear definition of ‘low-dose’ could lead to misinterpretations of preclinical findings, and thus hamper translation of these studies into the clinic.

This study was undertaken to provide some guidance on the MTD of chemotherapy in mice for future studies. We chose to use practical measurements that can be applied easily to any research setting, and which have been validated in previous studies [15]. By following this strategy, we found the MTD of some drugs to be quite different from commonly used dosages in the literature. The largest differences were seen with docetaxel and gemcitabine and to a lesser extent with 5-FU and irinotecan. Docetaxel had an MTD of 130 mg/kg, far higher than the commonly used dose of 16 to 33 mg/kg found in the literature [31–33]. The MTD of gemcitabine was 700 mg/kg, five-fold higher than those used by others (and our own group previously [34]) with the most common dose being 120 mg/kg [35–37]. It should also be noted however that many similar MTDs were found between this study and others. Cisplatin is commonly dosed at 5–6 mg/kg [34, 38, 39], which is concordant with the MTD of 6 mg/kg found in this study. Similarly, vinorelbine is often administered at 10 mg/kg [40–42], the same dose reported here.

We anticipate that other research groups may want to use the MTDs described here as a starting point for their own studies, potentially reducing the number of animals needed to optimize protocols. However, there are some limitations to our studies. Firstly, although we found that the MTD for both cisplatin and vinorelbine were similar for C57BL/6 J and BALB/c mice, the BALB/c mice did show slightly more weight loss for the tested chemotherapeutics. This suggests that care should be taken when transposing dosages between strains, including immunodeficient strains. Secondly, we determined the MTD when given as a single dose. Although we found that multiple cycles of vinorelbine at single-dose MTD was very well tolerated, this was not the case for cisplatin. A dose reduction was needed to maintain the weights of the animals when giving multiple dosages. This suggests that some further optimization steps will be needed when using the dosages as described, depending on the required scheduling regimen and the mouse strain used and possibly the emetogenicity of the chemotherapeutic [43]. Furthermore, small differences between research centres may affect either the response to or toxicity from chemotherapy. These include temperature [44], time of dosing [45], sex [46], microbiome [47], and age [48] which should all be considered before beginning experimental studies.

Of interest, some of the MTDs that we determined are very close to or sometimes even over the reported LD50 [49]. Explanations for this could be related to the above-mentioned factors, and particularly with the mouse strain used for determining the LD50 [49]. For example, the LD50 for irinotecan was originally determined in ICR mice, whereas we used BALB/c mice, which also in the primary paper reportedly could tolerate higher dosages of irinotecan, similar to C57BL/6 mice [16]. Similarly, we found that cisplatin could be safely dosed at 6 mg/kg, and that 1/3 mice lost more than 20% weight after 8 mg/kg, while the reported LD50 of cisplatin is 6.6 mg/kg [49]. However, this LD50 is based on studies in DBA mice [50]; in other strains, dosages as high as 18 mg/kg have been reported [51]. These data underscore the importance of considering mouse background when interpreting preclinical chemotherapy dosages.

The final aim of this study was to investigate the effect of two common supportive care agents, ondansetron and dexamethasone. Both drugs are used in cancer patients to reduce chemotherapy and radiotherapy-induced nausea and vomiting, with dexamethasone also used to maintain weight in some circumstances [52, 53]. The anti-emetic effect of dexamethasone, a synthetic glucocorticoid, is not well understood, although several mechanisms have been put forward, such as anti-inflammatory effects, normalisation of the hypothalamic–pituitary–adrenal axis, and effects on serotonin [54]. Indeed, we found that also in mice dexamethasone showed a dose-dependent effect on chemotherapy-induced weight loss, with the optimum dosage at 0.2 mg/kg, which is within the dose range that is used in patients in this context [55]. Ondansetron is a serotonin 5-HT_3_ receptor antagonist used for the treatment of chemotherapy-induced nausea [56]. Chemotherapeutics induce the release of serotonin in the small intestine, which binds 5-HT_3_ receptors and induces emesis. Ondansetron outcompetes serotonin, preventing receptor binding and therefore acting as an effective anti-emetic [57]. It is used to prevent nausea and vomiting after chemotherapy or radiotherapy in humans, but also in company animals such as dogs and cats [58]. Two 5-HT_3_ subunits have been identified in mice, namely A and B subunits while in humans there are five, A-E [59]. The primary binding of ondansetron to 5-HT_3_ is via the subunit A receptor [60] and so this provided some rational for investigation in our supportive care studies. However, our results show that ondansetron alone is ineffective in reducing cisplatin-induced weight loss or improving clinical condition in mice. This is perhaps not completely unexpected, as ondansetron primarily regulates the vomit reflex along with nausea [55]. Since rodents are not able to vomit, it might be expected that only an improved appetite, not decreased vomiting would result in reduced weight loss. However, surprisingly, we found that ondansetron abolished the beneficial effect of dexamethasone on preventing chemotherapy-induced weight loss in mice. This is striking since in the clinical setting it is common practice to combine ondansetron with dexamethasone as this combination is better at emetic control than ondansetron alone [21]. Our data suggest that this combination should not be used in mice for this indication.

## Conclusion

Together, these data constitute a resource for other researchers investigating cytotoxic chemotherapy in mice, using the identified MTDs as a starting point for their studies.

## Additional file

Additional file 1: Additional information regarding the clinical severity score used to determine wellbeing of the mice and a comprehensive list of the chemotherapeutic drugs used in this study. (PDF 82 kb)

## Abbreviations

5-FU: 5-fluorouracil
5-HT_3_: 5-hydroxytryptamine 3
CY: Cyclophosphamide
I.p.: Intraperitoneally
LD_50_: Lethal dose 50
MTD: Maximum tolerated dose
PBS: Phosphate buffered saline
SPF: Specific pathogen free
Treg: Regulatory T cell

## References

[1] Le Tourneau C, Lee JJ, Siu LL (2009). Dose escalation methods in phase I cancer clinical trials. J Natl Cancer Inst.

[2] Cook AM, Lesterhuis WJ, Nowak AK, Lake RA (2016). Chemotherapy and immunotherapy: mapping the road ahead. Curr Opin Immunol.

[3] Aston WJ, Fisher SA, Khong A, Mok C, Nowak AK, Lake RA, Lesterhuis WJ (2014). Combining chemotherapy and checkpoint blockade in thoracic cancer: how to proceed?. Lung Cancer Manag.

[4] Frei E, Canellos GP (1980). Dose: a critical factor in cancer chemotherapy. Am J Med.

[5] Hryniuk WM, Bush H. The importance of dose intensity in chemotherapy of metastatic breast cancer. J Clin Oncol. 1984;2(11)1281–8. 10.1200/JCO.1984.2.11.1281 6387060

[6] Lesterhuis WJ, Punt CJ, Hato SV, Eleveld-Trancikova D, Jansen BJ, Nierkens S, Schreibelt G, de Boer A, Van Herpen CM, Kaanders JH (2011). Platinum-based drugs disrupt STAT6-mediated suppression of immune responses against cancer in humans and mice. J Clin Invest.

[7] Viaud S, Flament C, Zoubir M, Pautier P, LeCesne A, Ribrag V, Soria JC, Marty V, Vielh P, Robert C (2011). Cyclophosphamide induces differentiation of Th17 cells in cancer patients. Cancer Res.

[8] Klement G, Baruchel S, Rak J, Man S, Clark K, Hicklin DJ, Bohlen P, Kerbel RS (2000). Continuous low-dose therapy with vinblastine and VEGF receptor-2 antibody induces sustained tumor regression without overt toxicity. J Clin Invest.

[9] Browder T, Butterfield CE, Kraling BM, Shi B, Marshall B, O'Reilly MS, Folkman J (2000). Antiangiogenic scheduling of chemotherapy improves efficacy against experimental drug-resistant cancer. Cancer Res.

[10] Ghiringhelli F, Larmonier N, Schmitt E, Parcellier A, Cathelin D, Garrido C, Chauffert B, Solary E, Bonnotte B, Martin F (2004). CD4+CD25+ regulatory T cells suppress tumor immunity but are sensitive to cyclophosphamide which allows immunotherapy of established tumors to be curative. Eur J Immunol.

[11] Ikezawa Y, Nakazawa M, Tamura C, Takahashi K, Minami M, Ikezawa Z (2005). Cyclophosphamide decreases the number, percentage and the function of CD25+ CD4+ regulatory T cells, which suppress induction of contact hypersensitivity. J Dermatol Sci.

[12] Lutsiak ME, Semnani RT, De Pascalis R, Kashmiri SV, Schlom J, Sabzevari H (2005). Inhibition of CD4(+)25+ T regulatory cell function implicated in enhanced immune response by low-dose cyclophosphamide. Blood.

[13] Ercolini AM, Ladle BH, Manning EA, Pfannenstiel LW, Armstrong TD, Machiels JP, Bieler JG, Emens LA, Reilly RT, Jaffee EM (2005). Recruitment of latent pools of high-avidity CD8(+) T cells to the antitumor immune response. J Exp Med.

[14] Nowak AK, Robinson BW, Lake RA (2003). Synergy between chemotherapy and immunotherapy in the treatment of established murine solid tumors. Cancer Res.

[15] Newell DR, Burtles SS, Fox BW, Jodrell DI, Connors TA (1999). Evaluation of rodent-only toxicology for early clinical trials with novel cancer therapeutics. Br J Cancer.

[16] Kunimoto T, Nitta K, Tanaka T, Uehara N, Baba H, Takeuchi M, Yokokura T, Sawada S, Miyasaka T, Mutai M (1987). Antitumor activity of 7-ethyl-10-[4-(1-piperidino)-1-piperidino]carbonyloxy-camptothec in, a novel water-soluble derivative of camptothecin, against murine tumors. Cancer Res.

[17] Watters JW, Kloss EF, Link DC, Graubert TA, McLeod HL (2003). A mouse-based strategy for cyclophosphamide pharmacogenomic discovery. J Appl Physiol (1985).

[18] Frick A, Fedoriw Y, Richards K, Damania B, Parks B, Suzuki O, Benton CS, Chan E, Thomas RS, Wiltshire T (2015). Immune cell-based screening assay for response to anticancer agents: applications in pharmacogenomics. Pharmgenomics Pers Med.

[19] Workman P, Aboagye EO, Balkwill F, Balmain A, Bruder G, Chaplin DJ, Double JA, Everitt J, Farningham DA, Glennie MJ (2010). Guidelines for the welfare and use of animals in cancer research. Br J Cancer.

[20] Kurhe Y, Mahesh R (2015). Ondansetron attenuates co-morbid depression and anxiety associated with obesity by inhibiting the biochemical alterations and improving serotonergic neurotransmission. Pharmacol Biochem Behav.

[21] Olver I, Paska W, Depierre A, Seitz JF, Stewart DJ, Goedhals L, McQuade B, McRae J, Wilkinson JR (1996). A multicentre, double-blind study comparing placebo, ondansetron and ondansetron plus dexamethasone for the control of cisplatin-induced delayed emesis. Ondansetron delayed emesis study group. Ann Oncol.

[22] Ottewell PD, Monkkonen H, Jones M, Lefley DV, Coleman RE, Holen I (2008). Antitumor effects of doxorubicin followed by zoledronic acid in a mouse model of breast cancer. J Natl Cancer Inst.

[23] Desai VG, Herman EH, Moland CL, Branham WS, Lewis SM, Davis KJ, George NI, Lee T, Kerr S, Fuscoe JC (2013). Development of doxorubicin-induced chronic cardiotoxicity in the B6C3F1 mouse model. Toxicol Appl Pharmacol.

[24] Eralp Y, Wang X, Wang JP, Maughan MF, Polo JM, Lachman LB (2004). Doxorubicin and paclitaxel enhance the antitumor efficacy of vaccines directed against HER 2/neu in a murine mammary carcinoma model. Breast Cancer Res.

[25] Johansen PB (1981). Doxorubicin pharmacokinetics after intravenous and intraperitoneal administration in the nude mouse. Cancer Chemother Pharmacol.

[26] Casares N, Pequignot MO, Tesniere A, Ghiringhelli F, Roux S, Chaput N, Schmitt E, Hamai A, Hervas-Stubbs S, Obeid M (2005). Caspase-dependent immunogenicity of doxorubicin-induced tumor cell death. J Exp Med.

[27] Ma Y, Aymeric L, Locher C, Mattarollo SR, Delahaye NF, Pereira P, Boucontet L, Apetoh L, Ghiringhelli F, Casares N (2011). Contribution of IL-17-producing gamma delta T cells to the efficacy of anticancer chemotherapy. J Exp Med.

[28] Yamazaki T, Hannani D, Poirier-Colame V, Ladoire S, Locher C, Sistigu A, Prada N, Adjemian S, Catani JP, Freudenberg M (2014). Defective immunogenic cell death of HMGB1-deficient tumors: compensatory therapy with TLR4 agonists. Cell Death Differ.

[29] Brode S, Cooke A (2008). Immune-potentiating effects of the chemotherapeutic drug cyclophosphamide. Crit Rev Immunol.

[30] Motoyoshi Y, Kaminoda K, Saitoh O, Hamasaki K, Nakao K, Ishii N, Nagayama Y, Eguchi K (2006). Different mechanisms for anti-tumor effects of low- and high-dose cyclophosphamide. Oncol Rep.

[31] Mason KA, Hunter NR, Milas M, Abbruzzese JL, Milas L (1997). Docetaxel enhances tumor radioresponse in vivo. Clin Cancer Res.

[32] Jia Y, Zhou D, Jia Q, Ying Y, Chen S (2016). Synergistic and attenuated effect of HSS in combination treatment with docetaxel plus cisplatin in human non-small-cell lung SPC-A-1 tumor xenograft. Biomed Pharmacother.

[33] Michalska M, Schultze-Seemann S, Bogatyreva L, Hauschke D, Wetterauer U, Wolf P (2016). In vitro and in vivo effects of a recombinant anti-PSMA immunotoxin in combination with docetaxel against prostate cancer. Oncotarget.

[34] Lesterhuis WJ, Salmons J, Nowak AK, Rozali EN, Khong A, Dick IM, Harken JA, Robinson BW, Lake RA (2013). Synergistic effect of CTLA-4 blockade and cancer chemotherapy in the induction of anti-tumor immunity. PLoS One.

[35] Vincent J, Mignot G, Chalmin F, Ladoire S, Bruchard M, Chevriaux A, Martin F, Apetoh L, Rebe C, Ghiringhelli F (2010). 5-fluorouracil selectively kills tumor-associated myeloid-derived suppressor cells resulting in enhanced T cell-dependent antitumor immunity. Cancer Res.

[36] Peters GJ, Bergman AM, Ruiz van Haperen VW, Veerman G, Kuiper CM, Braakhuis BJ (1995). Interaction between cisplatin and gemcitabine in vitro and in vivo. Semin Oncol.

[37] Suzuki E, Kapoor V, Jassar AS, Kaiser LR, Albelda SM (2005). Gemcitabine selectively eliminates splenic Gr-1+/CD11b+ myeloid suppressor cells in tumor-bearing animals and enhances antitumor immune activity. Clin Cancer Res.

[38] Silver DF, Piver MS (1999). Effects of recombinant human erythropoietin on the antitumor effect of cisplatin in SCID mice bearing human ovarian cancer: a possible oxygen effect. Gynecol Oncol.

[39] Shirasaka T, Shimamoto Y, Ohshimo H, Saito H, Fukushima M (1993). Metabolic basis of the synergistic antitumor activities of 5-fluorouracil and cisplatin in rodent tumor models in vivo. Cancer Chemother Pharmacol.

[40] Kruczynski A, Colpaert F, Tarayre JP, Mouillard P, Fahy J, Hill BT (1998). Preclinical in vivo antitumor activity of vinflunine, a novel fluorinated Vinca alkaloid. Cancer Chemother Pharmacol.

[41] Kraus-Berthier L, Jan M, Guilbaud N, Naze M, Pierre A, Atassi G (2000). Histology and sensitivity to anticancer drugs of two human non-small cell lung carcinomas implanted in the pleural cavity of nude mice. Clin Cancer Res.

[42] Bonfil RD, Russo DM, Binda MM, Delgado FM, Vincenti M (2002). Higher antitumor activity of vinflunine than vinorelbine against an orthotopic murine model of transitional cell carcinoma of the bladder. Urol Oncol.

[43] Hesketh PJ (1999). Defining the emetogenicity of cancer chemotherapy regimens: relevance to clinical practice. Oncologist.

[44] Kokolus KM, Capitano ML, Lee CT, Eng JW, Waight JD, Hylander BL, Sexton S, Hong CC, Gordon CJ, Abrams SI (2013). Baseline tumor growth and immune control in laboratory mice are significantly influenced by subthermoneutral housing temperature. Proc Natl Acad Sci U S A.

[45] Granda TG, D'Attino RM, Filipski E, Vrignaud P, Garufi C, Terzoli E, Bissery MC, Levi F (2002). Circadian optimisation of irinotecan and oxaliplatin efficacy in mice with Glasgow osteosarcoma. Br J Cancer.

[46] Clocchiatti A, Cora E, Zhang Y, Dotto GP (2016). Sexual dimorphism in cancer. Nat Rev Cancer.

[47] Iida N, Dzutsev A, Stewart CA, Smith L, Bouladoux N, Weingarten RA, Molina DA, Salcedo R, Back T, Cramer S (2013). Commensal bacteria control cancer response to therapy by modulating the tumor microenvironment. Science.

[48] Andriyanov AV, Portnoy E, Koren E, Inesa S, Eyal S, Goldberg SN, Barenholz Y (2017). Therapeutic efficacy of combined PEGylated liposomal doxorubicin and radiofrequency ablation: comparing single and combined therapy in young and old mice. J Control Release.

[49] ChemIDplus Advanced - Chemical information with searchable synonyms, structures, and formulas [http://chem.sis.nlm.nih.gov/chemidplus/]. Accessed Dec 2016.

[50] Gibson D, Gean KF, Ben-Shoshan R, Ramu A, Ringel I, Katzhendler J (1991). Preparation, characterization, and anticancer activity of a series of cis-PtCl2 complexes linked to anthraquinone intercalators. J Med Chem.

[51] Dorr RT, Soble MJ (1988). Cimetidine enhances cisplatin toxicity in mice. J Cancer Res Clin Oncol.

[52] Walton SM (2000). Advances in use of the 5-HT3 receptor antagonists. Expert Opin Pharmacother.

[53] Jantunen IT, Kataja VV, Muhonen TT (1997). An overview of randomised studies comparing 5-HT3 receptor antagonists to conventional anti-emetics in the prophylaxis of acute chemotherapy-induced vomiting. Eur J Cancer.

[54] Chu CC, Hsing CH, Shieh JP, Chien CC, Ho CM, Wang JJ (2014). The cellular mechanisms of the antiemetic action of dexamethasone and related glucocorticoids against vomiting. Eur J Pharmacol.

[55] Hesketh PJ (2008). Chemotherapy-induced nausea and vomiting. N Engl J Med.

[56] Wilde MI, Markham A (1996). Ondansetron. A review of its pharmacology and preliminary clinical findings in novel applications. Drugs.

[57] Aapro MS, Grunberg SM, Manikhas GM, Olivares G, Suarez T, Tjulandin SA, Bertoli LF, Yunus F, Morrica B, Lordick F (2006). A phase III, double-blind, randomized trial of palonosetron compared with ondansetron in preventing chemotherapy-induced nausea and vomiting following highly emetogenic chemotherapy. Ann Oncol.

[58] Santos LC, Ludders JW, Erb HN, Martin-Flores M, Basher KL, Kirch P (2011). A randomized, blinded, controlled trial of the antiemetic effect of ondansetron on dexmedetomidine-induced emesis in cats. Vet Anaesth Analg.

[59] Hassaine G, Deluz C, Grasso L, Wyss R, Tol MB, Hovius R, Graff A, Stahlberg H, Tomizaki T, Desmyter A (2014). X-ray structure of the mouse serotonin 5-HT3 receptor. Nature.

[60] Duffy NH, Lester HA, Dougherty DA (2012). Ondansetron and granisetron binding orientation in the 5-HT(3) receptor determined by unnatural amino acid mutagenesis. ACS Chem Biol.

[61] Bertino JR, Sawicki WL, Lindquist CA, Gupta VS (1977). Schedule-dependent antitumor effects of methotrexate and 5-fluorouracil. Cancer Res.

[62] Harrington EA, Bebbington D, Moore J, Rasmussen RK, Ajose-Adeogun AO, Nakayama T, Graham JA, Demur C, Hercend T, Diu-Hercend A (2004). VX-680, a potent and selective small-molecule inhibitor of the aurora kinases, suppresses tumor growth in vivo. Nat Med.

[63] Loupakis F, Cremolini C, Masi G, Lonardi S, Zagonel V, Salvatore L, Cortesi E, Tomasello G, Ronzoni M, Spadi R (2014). Initial therapy with FOLFOXIRI and bevacizumab for metastatic colorectal cancer. N Engl J Med.

[64] Guo H, Zhang Z, Su Z, Sun C, Zhang X, Zhao X, Lai X, Su Z, Li Y, Zhan JY (2016). Enhanced anti-tumor activity and reduced toxicity by combination andrographolide and bleomycin in ascitic tumor-bearing mice. Eur J Pharmacol.

[65] Marmor JB, Kozak D, Hahn GM (1979). Effects of systemically administered bleomycin or adriamycin with local hyperthermia on primary tumor and lung metastases. Cancer Treat Rep.

[66] Karimfar MH, Rostami S, Haghani K, Bakhtiyari S, Noori-Zadeh A (2015). MELATONIN ALLEVIATES BLEOMYCIN-INDUCED PULMONARY FIBROSIS IN MICE. J Biol Regul Homeost Agents.

[67] Pecorelli S, Wagenaar HC, Vergote IB, Curran D, Beex LV, Wiltshaw E, Vermorken JB (1999). Cisplatin (P), vinblastine (V) and bleomycin (B) combination chemotherapy in recurrent or advanced granulosa(−theca) cell tumours of the ovary. An EORTC gynaecological cancer cooperative group study. Eur J Cancer.

[68] Vermorken JB, Mesia R, Rivera F, Remenar E, Kawecki A, Rottey S, Erfan J, Zabolotnyy D, Kienzer HR, Cupissol D (2008). Platinum-based chemotherapy plus cetuximab in head and neck cancer. N Engl J Med.

[69] Drumond AL, Weng CC, Wang G, Chiarini-Garcia H, Eras-Garcia L, Meistrich ML (2011). Effects of multiple doses of cyclophosphamide on mouse testes: accessing the germ cells lost, and the functional damage of stem cells. Reprod Toxicol.

[70] Luznik L, Engstrom LW, Iannone R, Fuchs EJ (2002). Posttransplantation cyclophosphamide facilitates engraftment of major histocompatibility complex-identical allogeneic marrow in mice conditioned with low-dose total body irradiation. Biol Blood Marrow Transplant.

[71] Boulad F, Steinherz P, Reyes B, Heller G, Gillio AP, Small TN, Brochstein JA, Kernan NA, O'Reilly RJ (1999). Allogeneic bone marrow transplantation versus chemotherapy for the treatment of childhood acute lymphoblastic leukemia in second remission: a single-institution study. J Clin Oncol.

[72] Bear HD, Tang G, Rastogi P, Geyer CE, Robidoux A, Atkins JN, Baez-Diaz L, Brufsky AM, Mehta RS, Fehrenbacher L (2012). Bevacizumab added to neoadjuvant chemotherapy for breast cancer. N Engl J Med.

[73] Judson I, Verweij J, Gelderblom H, Hartmann JT, Schoffski P, Blay JY, Kerst JM, Sufliarsky J, Whelan J, Hohenberger P (2014). Doxorubicin alone versus intensified doxorubicin plus ifosfamide for first-line treatment of advanced or metastatic soft-tissue sarcoma: a randomised controlled phase 3 trial. Lancet Oncol.

[74] Johnson TS, Terrell CE, Millen SH, Katz JD, Hildeman DA, Jordan MB (2014). Etoposide selectively ablates activated T cells to control the immunoregulatory disorder hemophagocytic lymphohistiocytosis. J Immunol.

[75] Slater LM, Stupecky M, Sweet P, Osann K, Eklof A, Arquilla ER (2001). Etoposide induction of tumor immunity in Lewis lung cancer. Cancer Chemother Pharmacol.

[76] Hooker AM, Horne R, Morley AA, Sykes PJ (2002). Dose-dependent increase or decrease of somatic intrachromosomal recombination produced by etoposide. Mutat Res.

[77] Diehl V, Franklin J, Pfreundschuh M, Lathan B, Paulus U, Hasenclever D, Tesch H, Herrmann R, Dorken B, Muller-Hermelink HK (2003). Standard and increased-dose BEACOPP chemotherapy compared with COPP-ABVD for advanced Hodgkin's disease. N Engl J Med.

[78] Saida Y, Watanabe S, Tanaka T, Baba J, Sato K, Shoji S, Igarashi N, Kondo R, Okajima M, Koshio J (2015). Critical roles of Chemoresistant Effector and regulatory T cells in antitumor immunity after Lymphodepleting chemotherapy. J Immunol.

[79] Suzuki Y, Yuen S, Ashley R (2005). Short, thin asbestos fibers contribute to the development of human malignant mesothelioma: pathological evidence. Int J Hyg Environ Health.

[80] Oettle H, Neuhaus P, Hochhaus A, Hartmann JT, Gellert K, Ridwelski K, Niedergethmann M, Zulke C, Fahlke J, Arning MB (2013). Adjuvant chemotherapy with gemcitabine and long-term outcomes among patients with resected pancreatic cancer: the CONKO-001 randomized trial. JAMA.

[81] Ohdo S, Makinosumi T, Ishizaki T, Yukawa E, Higuchi S, Nakano S, Ogawa N (1997). Cell cycle-dependent chronotoxicity of irinotecan hydrochloride in mice. J Pharmacol Exp Ther.

[82] Choi SH, Tsuchida Y, Yang HW (1998). Oral versus intraperitoneal administration of irinotecan in the treatment of human neuroblastoma in nude mice. Cancer Lett.

[83] Guichard S, Chatelut E, Lochon I, Bugat R, Mahjoubi M, Canal P (1998). Comparison of the pharmacokinetics and efficacy of irinotecan after administration by the intravenous versus intraperitoneal route in mice. Cancer Chemother Pharmacol.

[84] Cunningham D, Humblet Y, Siena S, Khayat D, Bleiberg H, Santoro A, Bets D, Mueser M, Harstrick A, Verslype C (2004). Cetuximab monotherapy and cetuximab plus irinotecan in irinotecan-refractory metastatic colorectal cancer. N Engl J Med.

[85] Winton T, Livingston R, Johnson D, Rigas J, Johnston M, Butts C, Cormier Y, Goss G, Inculet R, Vallieres E (2005). Vinorelbine plus cisplatin vs. observation in resected non-small-cell lung cancer. N Engl J Med.

