# Supplementary material for: A systematic investigation of the maximum tolerated dose of cytotoxic chemotherapy with and without supportive care in mice
Source: BMC Cancer. 2017 Oct 16;17:684. doi: 10.1186/s12885-017-3677-7 (PMC5644108; doi:10.1186/s12885-017-3677-7)
Supplement: Additional file 1: — Additional information regarding the clinical severity score used to determine wellbeing of the mice and a comprehensive list of the chemotherapeutic drugs used in this study. (PDF 82 kb) [file 12885_2017_3677_MOESM1_ESM.pdf]

ADDITIONAL FILE

A Systematic Investigation of the Maximum Tolerated Dose of Cytotoxic Chemotherapy with and without Supportive Care in Mice

Wayne J. Aston[1,2], Danika E. Hope[1,2], Anna K. Nowak[1,2,3], Bruce W. Robinson[1,2], Richard A. Lake[1,2], W. Joost Lesterhuis[1,2*]

[1]National Centre for Asbestos Related Diseases, [2]Faculty of Health and Medical Sciences, The University of Western Australia, Nedlands WA 6009, Australia.

[3]Department of Medical Oncology, Sir Charles Gairdner Hospital, Nedlands, WA, 6009, Australia.

Key words: chemotherapy, mice, dose optimization, maximum tolerated dose, MTD, supportive care, cancer.

Short title: cytotoxic chemotherapy dose optimization in mice

***Corresponding author**: W. Joost Lesterhuis, National Centre for Asbestos Related Diseases, Faculty of Health and Medical Sciences, University of Western Australia, 5th Floor, QQ Block, 6 Verdun Street, Nedlands WA 6009, Australia; Phone: (+61) 8 6151 0895, Fax: (+61) 8 6151 1027; Email: willem.lesterhuis@uwa.edu.au

Score		0	1	2
Appearance	Weight loss	Steady weight	> 10% weight loss	15%-19.9% weight loss
	Coat	Normal	Mild ruffled coat	Moderate ruffled coat, ungroomed
	Body condition	Normal	Thin	Loss of body fat, failure to grow
	Body Posture	Normal	Hunched	Hunched and still
	Movement	Normal	Reduced/slow	Reluctant to move when touched
Activity	Proximity to others	Close contact	Somewhat separate	Completely separate
Other	Injection site	Normal	Some redness at margins	Redness and swelling

Supplementary Table S1: Clinical Severity Score. Parameters used for the clinical score determination of mice.

Chemotherapy	Supplier	Diluent
5-Fluorouracil	Fluorouracil Ebewe, Sandoz PTY LTD, Pyrmont NSW Australia	0.9% NaCl
Bleomycin	DBL Bleomycin Sulfate, Hospira, Mulgrave VIC Australia	Water for injection
Cisplatin	DBL Cisplatin, Hospira, Mulgrave VIC Australia	0.9% NaCl
Cyclophosphamide	Endoxan, Baxter, Toongabbie NSW Australia	0.9% NaCl
Docetaxel	Oncotaxel 80, Actavis, North Adelaide SA Australia	0.9% NaCl
Doxorubicin	Adriamycin, Pfizer, West Ryde NSW Australia	0.9% NaCl
Etoposide Phosphate	Etopophos, Bristol-Myers-Squibb, Mulgrave VIC Australia	Water for injection
Gemcitabine	DBL Gemcitabine, Hospira, Mulgrave VIC Australia	0.9% NaCl
Irinotecan	Irinotecan Actavis, Actavis, North Adelaide SA Australia	0.9% NaCl
Vinorelbine	Navelbine, Pierre Fabre Medicament, North Ryde NSW Australia	0.9% NaCl

Supplementary Table S2: Chemotherapeutics used in this study with corresponding supplier and diluent.
